# Supplementary material for: Mechanisms governing the pioneering and redistribution capabilities of the non-classical pioneer PU.1
Source: Nat Commun. 2020 Jan 21;11:402. doi: 10.1038/s41467-019-13960-2 (PMC6972792; doi:10.1038/s41467-019-13960-2)
Supplement: Supplementary file 7 — Source data [file 41467_2019_13960_MOESM7_ESM.zip › Source_Data/FigureS2/FigureS2B_MotifScanOutput/KmeansCluster4/homerResults/motif1.similar.html]

motif1

## Information for motif1

A
C
G
T
A
T
G
C
C
T
G
A
A
T
G
C
A
C
G
T
A
C
G
T
A
G
T
C
A
G
T
C
G
A
C
T
A
G
T
C
G
C
A
T
G
A
C
T
G
C
A
T
A
G
C
T
  
Reverse Opposite:  

T
C
G
A
C
G
T
A
C
T
G
A
C
G
T
A
T
C
A
G
C
T
G
A
C
T
A
G
A
C
T
G
C
G
T
A
C
G
T
A
T
A
C
G
G
A
C
T
T
A
C
G
T
G
C
A
  

|  |  |
| --- | --- |
| p-value: | 1e-2368 |
| log p-value: | -5.453e+03 |
| Information Content per bp: | 1.589 |
| Number of Target Sequences with motif | 3955.0 |
| Percentage of Target Sequences with motif | 87.60% |
| Number of Background Sequences with motif | 5541.8 |
| Percentage of Background Sequences with motif | 13.26% |
| Average Position of motif in Targets | 101.5 +/- 32.2bp |
| Average Position of motif in Background | 100.1 +/- 73.7bp |
| Strand Bias (log2 ratio + to - strand density) | -0.1 |
| Multiplicity (# of sites on avg that occur together) | 1.22 |
| Motif File: | file (matrix) reverse opposite |

### Similar de novo motifs found

|  |  |  |  |  |  |  |  |
| --- | --- | --- | --- | --- | --- | --- | --- |
| Rank | Match Score | Redundant Motif | P-value | log P-value | % of Targets | % of Background | Motif file |
| 1 | 0.976 | A G T C C G T A A T G C A C G T A C G T A G T C A G T C A G C T A G T C G C A T G A C T G A C T | 1e-2142 | -4934.053488 | 91.63% | 19.15% | motif file (matrix) |
| 2 | 0.972 | C T G A C G T A T C A G C T G A C T A G A C T G C G T A C G T A T A C G G C A T T A C G | 1e-2140 | -4927.865775 | 86.91% | 15.33% | motif file (matrix) |
| 3 | 0.960 | C A G T C G A T C G T A C G A T C T A G C T G A C T A G C T A G C G T A C G T A T A C G G C A T C T A G | 1e-1963 | -4520.447692 | 78.87% | 12.05% | motif file (matrix) |
| 4 | 0.953 | C T G A C G T A T C A G C T G A C T A G A C T G C G T A C G T A T A C G A C G T | 1e-1836 | -4229.352898 | 78.18% | 13.06% | motif file (matrix) |
| 5 | 0.908 | T C A G C T G A A C T G A C T G C G T A C G T A T A C G G C A T T A C G | 1e-1732 | -3989.789333 | 77.52% | 13.89% | motif file (matrix) |
| 6 | 0.942 | C T G A A T G C G A C T A C G T A G T C G A T C A G T C G A T C G A C T G C A T G A C T | 1e-1716 | -3951.905599 | 83.41% | 18.26% | motif file (matrix) |
| 7 | 0.935 | A T G C G C T A A T G C G A C T A G C T A G T C G A T C G A C T A G T C G A C T G A C T G A C T A G C T | 1e-1706 | -3929.586561 | 77.14% | 13.98% | motif file (matrix) |
| 8 | 0.940 | C T G A A T G C C G A T C G A T G T A C A G T C G A C T A G T C G C A T G C A T | 1e-1569 | -3614.003077 | 95.17% | 32.94% | motif file (matrix) |
| 9 | 0.934 | C T A G C T A G C T A G T C A G C G T A G C T A T A C G A G C T C T A G T A G C | 1e-1553 | -3576.417118 | 73.36% | 13.55% | motif file (matrix) |
| 10 | 0.914 | T C A G C T G A A C T G A C T G C G T A C G T A T A C G G C A T | 1e-1537 | -3539.701739 | 87.02% | 24.27% | motif file (matrix) |
| 11 | 0.945 | C G T A T C G A C T G A A C T G A C T G C G T A C G T A A T C G A G C T | 1e-1517 | -3495.099719 | 79.71% | 18.39% | motif file (matrix) |
| 12 | 0.908 | G A T C T C G A A G T C G C A T C G A T G T A C A G T C G A C T G A T C A T G C G A C T | 1e-1417 | -3264.243695 | 69.46% | 12.96% | motif file (matrix) |
| 13 | 0.901 | C G T A C G T A C G A T T A C G C G T A C T A G T C A G T G C A C G T A T A C G G A C T C G T A C T G A C T G A | 1e-1355 | -3120.266862 | 83.03% | 23.90% | motif file (matrix) |
| 14 | 0.877 | C T G A C G T A C G T A T C A G T G C A T C A G T C A G G C T A C G T A T A C G | 1e-1263 | -2909.384038 | 79.89% | 22.87% | motif file (matrix) |
| 15 | 0.875 | C T A G C T A G A C T G C T G A C G T A T C A G A G C T | 1e-1236 | -2847.075639 | 68.46% | 14.90% | motif file (matrix) |
| 16 | 0.872 | A T G C C G T A A G T C A C G T A C G T A G T C A G T C | 1e-1233 | -2840.577761 | 56.90% | 8.54% | motif file (matrix) |
| 17 | 0.915 | C T G A C T G A C T A G C T A G T G C A C T G A T A C G C G A T C A T G T C A G G C T A C G A T | 1e-1188 | -2736.892638 | 63.79% | 12.75% | motif file (matrix) |
| 18 | 0.840 | A C T G C T G A C T A G A C T G C G T A C T G A A T C G | 1e-925 | -2131.436765 | 58.85% | 13.84% | motif file (matrix) |
| 19 | 0.809 | C G T A C G T A A T C G T G C A C T A G A C T G C G T A C G T A A T C G | 1e-871 | -2007.367780 | 50.19% | 9.78% | motif file (matrix) |
| 20 | 0.796 | G C T A C G T A T A C G T C A G T C A G A C T G T C G A T C G A | 1e-724 | -1669.038392 | 69.77% | 26.12% | motif file (matrix) |
| 21 | 0.815 | C A G T C T A G T C G A T C A G A C T G G T C A G T C A A T C G | 1e-714 | -1644.565586 | 53.95% | 14.63% | motif file (matrix) |
| 22 | 0.807 | T C G A G T C A C T G A C T A G C T A G C T A G A C T G C G T A C G T A | 1e-644 | -1483.310081 | 67.77% | 26.60% | motif file (matrix) |
| 23 | 0.877 | C G A T A G C T C G A T C T A G C T G A C T A G A C T G C G T A C G T A T A C G G A C T C G A T | 1e-315 | -727.306557 | 26.69% | 6.83% | motif file (matrix) |
| 24 | 0.783 | T C G A C G T A A C T G C T G A A C T G A C T G C G T A C G T A A T C G T G A C G C T A C T G A | 1e-216 | -497.728595 | 23.52% | 7.35% | motif file (matrix) |
| 25 | 0.790 | C T A G A G C T A C G T G A C T A T G C A G T C A G T C A G T C | 1e-208 | -480.791837 | 28.93% | 10.93% | motif file (matrix) |
| 26 | 0.751 | C T G A C T G A C T G A T A C G C T G A C T A G C A T G T C G A C G T A T C A G T A G C G T C A C T G A | 1e-206 | -476.271497 | 14.64% | 2.93% | motif file (matrix) |
| 27 | 0.796 | C G T A A C T G A C G T A C G T A G T C A G T C A G T C | 1e-178 | -410.343825 | 53.07% | 31.38% | motif file (matrix) |
| 28 | 0.819 | A G T C C T G A A C T G A C G T A C G T A G T C A G T C | 1e-163 | -376.518518 | 27.26% | 11.40% | motif file (matrix) |
| 29 | 0.779 | C T G A A C T G A C T G T C A G C T A G C T G A T C G A T A C G A C G T A C T G G T A C | 1e-122 | -282.880457 | 5.71% | 0.60% | motif file (matrix) |
| 30 | 0.790 | C G T A A C T G A C G T A C G T A G T C A G T C A G C T C A G T | 1e-67 | -155.241376 | 11.25% | 4.46% | motif file (matrix) |
